# Brain glutamate and gamma-aminobutyric acid levels across COVID-19 lockdowns in patients with recurrent major depressive disorder and healthy individuals

**DOI:** 10.1038/s41598-025-05734-2

**Published:** 2025-07-01

**Authors:** Valentin Popper, Benjamin Spurny-Dworak, Jakob Unterholzner, Murray Reed, Theresa Wechsler, Alexander Kautzky, Peter Stöhrmann, Manfred Klöbl, Andreas Mühlberger, Richard Frey, Dan Rujescu, Rupert Lanzenberger, Thomas Vanicek

**Affiliations:** 1 Department of Psychiatry and Psychotherapy, Medical University of Vienna, Vienna, Austria; 2 Comprehensive Center for Clinical Neurosciences and Mental Health, Medical University of Vienna, Vienna, Austria; 3 Department for Psychology, Clinical Psychology and Psychotherapy, University of Regensburg, Regensburg, Germany

**Keywords:** Neuroscience, Depression

## Abstract

The coronavirus disease 2019 (COVID-19) led to substantial social restriction measures. Social isolation has been demonstrated to promote psychiatric symptoms and to dysregulate gamma-aminobutyric acid (GABA) and glutamate levels. The aim of this investigation was to observe brain GABA and glutamate concentrations and depressive symptom severity in association to lockdowns in patients with recurrent major depression disorder (rMDD) and healthy individuals (HI). In this longitudinal study, 18 patients with rMDD (11 female: 37.0 ± 10.0years) and 28 HI (16 female, 28.1 ± 5.0years) underwent three magnetic resonance spectroscopy imaging (MRSI) measurements over multiple lockdowns. Ratios of GABA+ (GABA + macromolecules) and glutamate + glutamine (Glx) to total creatinine (tCr) as well as GABA+/Glx ratios were calculated for subcortical regions and the insula. Depressive symptom severity and social support were assessed at each visit. Lockdowns did not significantly change neurotransmitter ratios in individual brain regions (all *p*_corrected_ > 0.05). Further, no significant changes in Beck’s Depression Inventory II (BDI-II) scores occurred along the lockdowns (all *p*_corrected_ > 0.05). Our results may be explained by ceiling effects of the beginning of the pandemic and the first lockdown, by good social support during the pandemic in HI and a small sample size. Patients with rMDD reported an insufficient social support, suggesting a special vulnerability to social isolation due to pandemics.

**Supplementary Information:**

The online version contains supplementary material available at 10.1038/s41598-025-05734-2.

## Introduction

The coronavirus disease 2019 (COVID-19) is a serious condition with potentially long-lasting negative effects on physical and mental health^1^. To inhibit the spread of the virus, most governments introduced social restriction measures^2^. The pandemic, and in particular, social isolation due to distancing rules, quarantines, and lockdowns, were reported to have adverse psychological effects and increase the incidence of mental disorders^3^. Online surveys in India and Japan reported increased loneliness during the COVID-19 pandemic^4,5^, which is associated with the onset of depressive symptoms^6^.

Preclinical studies demonstrated a relationship between the number of social contacts and brain structure^7^. Social restriction measures due to the COVID-19 pandemic led to strongly reduced access to peer groups and overall limited contacts, which could have had neurobiological effects. Notably, a study on participants of a 14-month Antarctic expedition reported a decline in the gray matter volume (GMV)^8^. In addition to brain structural changes, alterations in brain function due to social isolation are of scientific interest. Glutamate and gamma-aminobutyric acid (GABA) are important excitatory and inhibitory neurotransmitters in the mammalian brain. Investigations described an association of social isolation and altered responsiveness of GABA receptors^9^. Further, socially isolated rats were found to have attenuated glutamate and glutamine levels in the dorsal hippocampus^10^. Changing environmental conditions may be capable of influencing GABAergic and glutamatergic transmission, as acute stress and pharmacological interventions were found to alter neurotransmission in humans^11,12^. Taken together, these findings suggest that the COVID-19 pandemic and social restriction measures as lockdowns might affect brain measures and in particular total levels of GABA and glutamate. Magnetic resonance spectroscopy imaging (MRSI) studies reported reduced glutamate and GABA levels in different brain regions in patients with depression^13^. Further, different effects of the COVID-19 pandemic on anxiety- and stress-levels and depressive symptoms in patients with depression compared to healthy individuals (HI) were reported^14^.

We aimed to elucidate the effects of lockdowns during the COVID-19 pandemic in Austria on GABA and glutamate levels, and changes in depressive symptoms. We hypothesized that lockdowns and the resulting social isolation disturb excitatory and inhibitory neurotransmission, represented in changes in total GABA and glutamate content, as well as in increases in depressive symptoms. For that purpose, we assessed GABA and glutamate levels in subcortical brain regions and the insula in association with lockdowns. We further hypothesized, that compared to HI, patients with recurrent Major Depressive Disorder (rMDD) would experience a greater impact of COVID-19 and social restriction measures on neurotransmitter ratios and depressive symptoms.

## Methods

### Study design

This longitudinal, quasi-experimental MRSI (magnetic resonance spectroscopy imaging) study was conducted on patients with rMDD and HI to measure GABA + (a combination of GABA and macromolecules) and Glx (glutamate + glutamine) concentrations in four subcortical brain regions (hippocampus, pallidum, putamen and thalamus) and the insula along different lockdowns in Austria. The study included a screening visit, three MRSI measurements, and a final examination. The first MRSI measurements (MRSI-1) were performed between September and November 2020, in the recovery phase of the first lockdown, when major restrictions had been lifted for several months, and right before the second lockdown. The second and third MRSI measurements (MRSI-2, MRSI-3) were performed immediately after strict lockdowns. The authors assert that all procedures contributing to this work comply with the ethical standards of the relevant national and institutional committees on human experimentation and with the Helsinki Declaration of 1975, as revised in 2008. All procedures involving human subjects/patients were approved by the Ethics Committee of the Medical University of Vienna (EK-Nr. 1410/2020).

### Lockdowns and social restriction measures in Austria

**Fig. 1:**
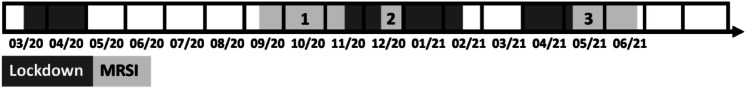
Timeline, showing lockdown periods due to COVID-19 pandemic in Austria (dark) and magnetic resonance spectroscopy imaging (MRSI) measurement periods (light). 1 = MRSI-1, 2 = MRSI-2, 3 = MRSI-3.

In Austria, strict lockdowns included a general curfew for the entire population except for work-related and medical reasons as well as the acquisition of food and goods for daily use. During light lockdowns, curfews were established for specific time frames and most businesses remained open; instead, social events (except funerals) were canceled, and distance learning was implemented by educational establishments. Tourism companies and restaurants were closed under strict and light lockdowns. The first strict lockdown (from 16th March to 1st May 2020) was followed by a series of strict and light lockdowns from November 2020 to December 2020 (strict lockdown from 16th November to 6th December) and from December 2020 to February 2021. Subsequently, there was another strict lockdown from 1st April to 2nd May 2021 in the capital of Austria, Vienna, with the same rules as for the previous lockdowns. For an overview of the lockdowns and measurement dates, see Fig. 1.

### Study collective

This study included 51 participants (21 patients with rMDD and 30 HI) who had been enrolled in previous studies by our group (ClinicalTrials.gov Identifier: NCT02810717, EK: 1761/2015 and ClinicalTrials.gov Identifier: NCT02753738, EK: 1739/2016.). The current research is independent from the previous performed studies. The health status was assessed through clinical examinations, comprising standard physical status of heart, lung, abdomen, bones, skin as well as vascular and neurologic status, electrocardiograms, and routine laboratory tests. A psychiatrist administered the Structured Clinical Interview for DSM-IV Axis-I Disorders to confirm that HI were free of mental disorders and patients with rMDD had experienced MDD symptoms of sufficient severity to meet diagnostic criteria. The status of recurrence was determined based on the medical history. Urine drug and pregnancy tests were administered at the screening visit and before each MRSI measurement to guarantee that participants did not meet exclusion criteria (substance abuse) and to avoid measurements with pregnant women. Due to a gap in knowledge about the effects of 3-Tesla magnetic resonance imaging (MRI) on pregnancy, we determined pregnancy as a relative contraindication for 3-Tesla MRI studies. The exclusion criteria were as follows: substance abuse, somatic illnesses, and any contraindications to MRI. At the screening visit, the participants provided written consent to participate in the study.

### Magnetic resonance spectroscopy imaging

MRI measurements were performed using a 3 Tesla MR Magnetom Prisma system (Siemens Medical, Erlangen, Germany) with a 64-channel head coil at the High-field MR Center, Department of Biomedical Imaging and Image-guided Therapy, Medical University of Vienna. We applied MRSI to detect transmitter changes in several brain regions, including multiple subcortical brain regions and the insula. Also, MRSI sequence enabled both the quantification of GABA+ and Glx. For volume of interest (VOI) placement and subsequent mask extraction for region-of-interest (ROI)-based MRSI analysis, a T1-weighted sequence (echo time (TE) = 1800 ms, repetition time (TR) = 2.37 ms, 208 slices, 288 × 288 matrix size, voxel size 1.15 × 1.15 × 0.85 mm) was performed before each MRSI scan.

**Fig. 2:**
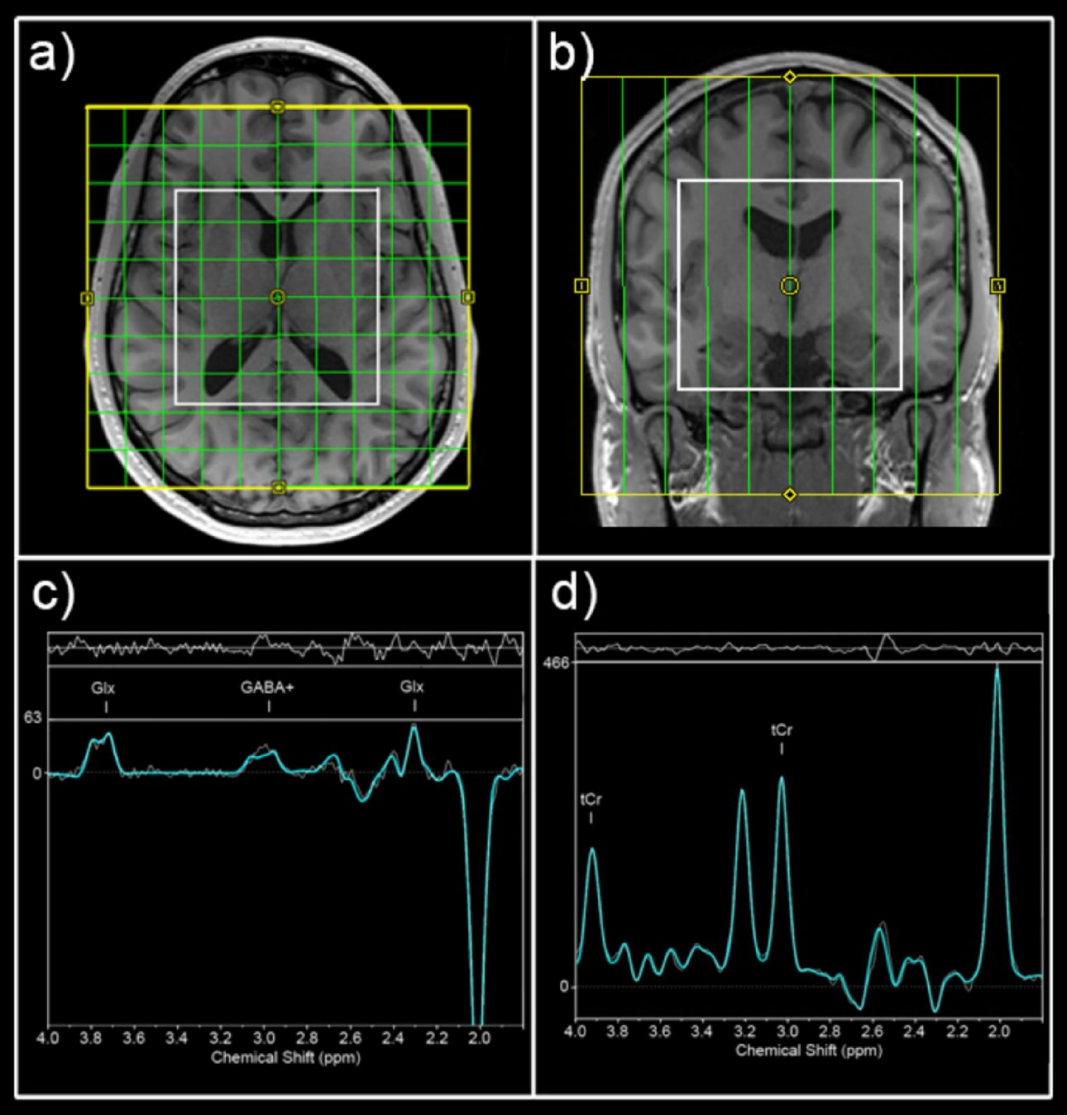
Field of view position and exemplary spectra. Field of view (yellow) and volume of interest (white) are shown in sagittal (**a**) and horizontal (**b**) view. Exemplary difference (**c**) and edit-off spectra (**d**) are presented showing peaks of Glx (glutamate + glutamine), GABA + (GABA + macromolecules) and total creatine (tCr).

Regarding the spectroscopic data, we used a spiral-encoded 3D GABA-edited Mescher-Garwood-localized adiabatic selective refocusing (MEGA-LASER) MRSI sequence (TE = 68 ms), which included real-time correction for rigid-body motion bias; moreover, the center frequency was applied using volumetric, dual-contrast, echo planar imaging-based navigators with a repetition time of 1.6 s and updates at 3.2-s intervals, as previously described^15,16^. The VOI was placed parallel to the anterior–posterior commissure line with a VOI of 80 (left–right) × 90 (anterior–posterior) × 80 (superior-inferior) mm^3^ and a field of view (FOV) of 160 × 160 × 160 mm^3^ (see Fig. 2). The acquired matrix size of 10 × 10 × 10 (i.e., ~ 4 cm^3^ nominal voxel size) was interpolated to a 16 × 16 × 16 matrix (i.e., ~ 1 cm^3^ nominal voxel size) during spectral processing. MEGA-editing pulses using 60-Hz Gaussian pulses with a duration of 14.8 ms were set to 1.9 ppm during EDIT-ON acquisition. We acquired 32 acquisition-weighted averages, which employed two-step phase cycling for a total scan time of 15:09 min. An advanced Siemens shimming procedure with manual adjustments was performed before each MRSI scan.

### Imaging data analysis

For MRSI data analysis, we used a combination of MATLAB (R2013a, MathWorks, Natick, MA, USA), Bash (4.2.25, Free Software Foundation, Boston, MA, USA), MINC (2.0, MINC Tools, McConnell Brain Imaging Center, Montreal, QC, Canada), and LCModel software (6.3–1, S. Provencher, LCModel, Oakville, ON, Canada). The GAMMA library was used to create two different basis sets, i.e., one for the non-edited spectra (containing 21 brain metabolites, including total creatine [tCr]) and the other for the difference spectrum (containing GABA+, Glx, etc.)^17^. Cramér–Rao lower bound (CRLB) thresholds were set at 30%; additionally, the spectra were visually inspected. Region of interest (ROI)-based quantification (described in the study by Spurny et al.^18^) was used to analyze neurotransmitter ratios in the hippocampus, insula, putamen, pallidum, and thalamus. FreeSurfer 7.1 software was used for automated segmentation of structural images and mask extraction; furthermore, spectral maps of GABA+, Glx, and tCr were interpolated to the structural image resolution (288 × 288 × 208) and overlaid with the derived masks, followed by the calculation of the mean GABA+/tCr and Glx/tCr ratios within each ROI. Thereby, spectral data of solely grey matter content was evaluated within each mask. We excluded ROIs with < 90% valid voxels based on the CRLB thresholds.

### Assessment of depressive symptoms, restriction measures and social support

To investigate the severity of depressive symptoms, all participants completed the Beck’s Depression Inventory-II (BDI-II) at the screening visit and at each measurement^19^. Stringency index, a tool recently developed by the University of Oxford to quantify the daily amount of governmental imposed restriction was used within this study^20^. We calculated a mean stringency index between measurements for each participant separately. The assessment furthermore comprised the Enriched Social Support Inventory (ESSI)^21^ to assess the participants’ social support at the measurements during the pandemic. Levels of 18 points and more in ESSI were considered good social support^21^. An online platform (EvaSys V8.0, Electric Paper Evaluationssysteme GmbH, Lüneburg, Germany) was used for applying the questionnaire.

### Statistical analysis

Descriptive statistics were calculated; moreover, between-group comparisons of age and sex were performed using t-tests and chi-square tests. To assess alterations in glutamate and GABA concentrations and differences between rMDD and HI, we estimated linear mixed models for each neurotransmitter ratio separately (i.e., GABA+/tCr, GABA+/Glx, and Glx/tCr). Time-point (3-levelled), group (patients with rMDD, HI), and ROIs (5-levelled) were assigned as fixed effects, neurotransmitter ratios as dependent variable and subject as the random intercept. Age (mean-centered by group) and sex were included as covariates. Then, linear mixed models were calculated for groups and ROIs separately. The significance level was set at *p* < 0.05. All mixed model results were corrected for multiple comparison using the Bonferroni procedure with a correction factor of 3 for pooled analyses, 6 for pooled group analyses, 15 for ROI-based analyses and 30 in ROI-based group analyses. Post-hoc tests were additionally corrected for multiple testing with a correction factor of 3 for the number of time-point comparisons. To evaluate changes in symptom severity and social support as well as differences between rMDD and HI, linear mixed models were calculated for the dependent variables BDI-II and ESSI with time and group as fixed factor and subject as the random intercept. Age (mean-centered) and sex were included as covariates again. Results were corrected for multiple comparison using the Bonferroni procedure with a correction factor of 12 (for the number of variables, groups, and measurements). To furthermore assess changes in governmental inflicted social restriction measures another linear mixed model was conducted for the stringency index. The results were corrected for multiple comparison using the Bonferroni procedure with a correction factor 4 for the number of dependent variables tested. If rendered significant, post-hoc tests were additionally corrected for multiple testing with a correction factor of 3. Correlation analyses were performed to investigate possible associations between neurotransmitter ratios (GABA+/tCr, GABA+/Glx, or Glx/tCr) and BDI-II for each time point. A correction factor of 9 for the number of time-point comparison x the number of neurotransmitter ratios was applied for correction for multiple testing. Statistical analyses were performed using SPSS Statistics (v25, 2010; SPSS, Inc., IBM Company, Chicago, United States).

## Results

Eighteen patients with rMDD (11 female by biological sex, mean age in years ± SD: 37.0 ± 10.0) and 28 HI (16 female by biological sex, 28.1 ± 5.0) were included in the analyses. Two patients with rMDD were excluded due to remission of depressive symptoms at screening visit and two HI were excluded due to clinically significant depressive symptoms at the screening visit or poor data quality, based on CRLB-threshold. Another patient of the rMDD group was excluded due to contraindications for MRI. There were no significant between-group differences in sex (chi-square test: *p* = 0.790); however, there was a significant between-group difference in age (t-test: *p* = 0.001). Therefore, age was mean-centered by group when included as a covariate. In the rMDD group, 7 patients had comorbidities, whereas 2 patients had anxiety disorders, 4 patients had personality disorders, and 4 patients suffered from a disturbance of activity and attention. For detailed descriptive statistics see Table 1 and Supplementary Table 1 and 2.

**Table 1:** Demographic information and psychological assessment are given as mean (± standard deviation, SD) in the whole collective as well as in each group.

	Whole collective	rMDD	HI	*p*-Values
Age (± SD)	31.59 (± 8.51)	37.00 (± 10.03)	28.11 (± 5.04)	0.001
Sex (%)	19 male (41.3%), 27 female (58.7%)	7 male (38.9%), 11 female (61.1%)	12 male (42.9%), 16 female (57.1%)	> 0.05
BDI-II 1		29.61 (± 9.87)	5.37 (± 5.02)	< 0.001
BDI-II 2		29.83 (± 10.89)	4.96 (± 5.85)	< 0.001
BDI-II 3		25.56 (± 15.42)	3.68 (± 5.41)	< 0.001
Medication		17/18	0/28	
ESSI 0	19.5 (± 5.5)	16.8 (± 4.7)	21.3 (± 5.3)	< 0.001
ESSI 1	19.1 (± 6.0)	15.3 (± 5.2)	21.6 (± 5.3)	< 0.001
ESSI 2	18.8 (± 6.4)	15.7 (± 4.6)	20.8 (± 6.7)	0.008
ESSI 3	17.8 (± 7.8)	15.7 (± 6.0)	19.1 (± 8.7)	> 0.05
Stringency 1	50.0 (± 0.6)	50.4 (± 0.4)	49.7 (± 0.5)	
Stringency 2	68.7 (± 6.2)	65.2 (± 6.6)	71.1 (± 4.6)	
Stringency 3	77.0 (± 0.5)	77.2 (± 0.4)	76.8 (± 0.6)	

rMDD, recurrent major depressive disorder; HI, healthy individuals; BDI-II, Beck’s depression inventory II; ESSI, enriched social support inventory; stringency, stringency index, MRSI, magnetic resonance spectroscopy imaging; 0, screening visit, 1, MRSI-1, 2, MRSI-2, 3, MRSI-3. ESSI cut-off for good social support is 18 points.

### Social restriction measures and neurotransmitter ratios

**Fig. 3:**
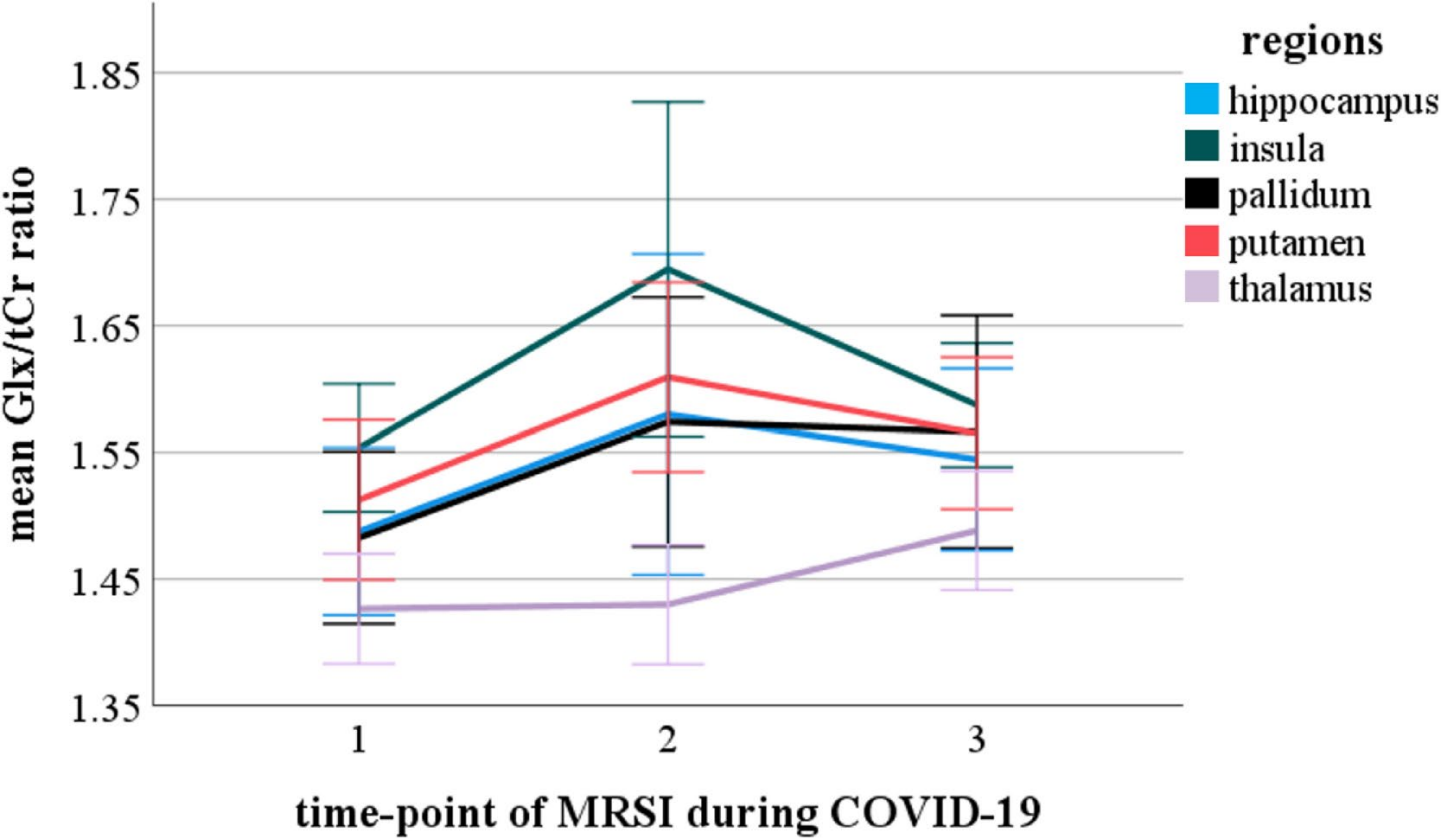
Mean Glx/tCr ratios for the hippocampus, insula, thalamus, pallidum, and putamen in the whole collective during the COVID-19 pandemic. Error bars indicating SD. Glx/tCr, glutamate + glutamine/total creatinine; SD, standard deviation.

**Fig. 4:**
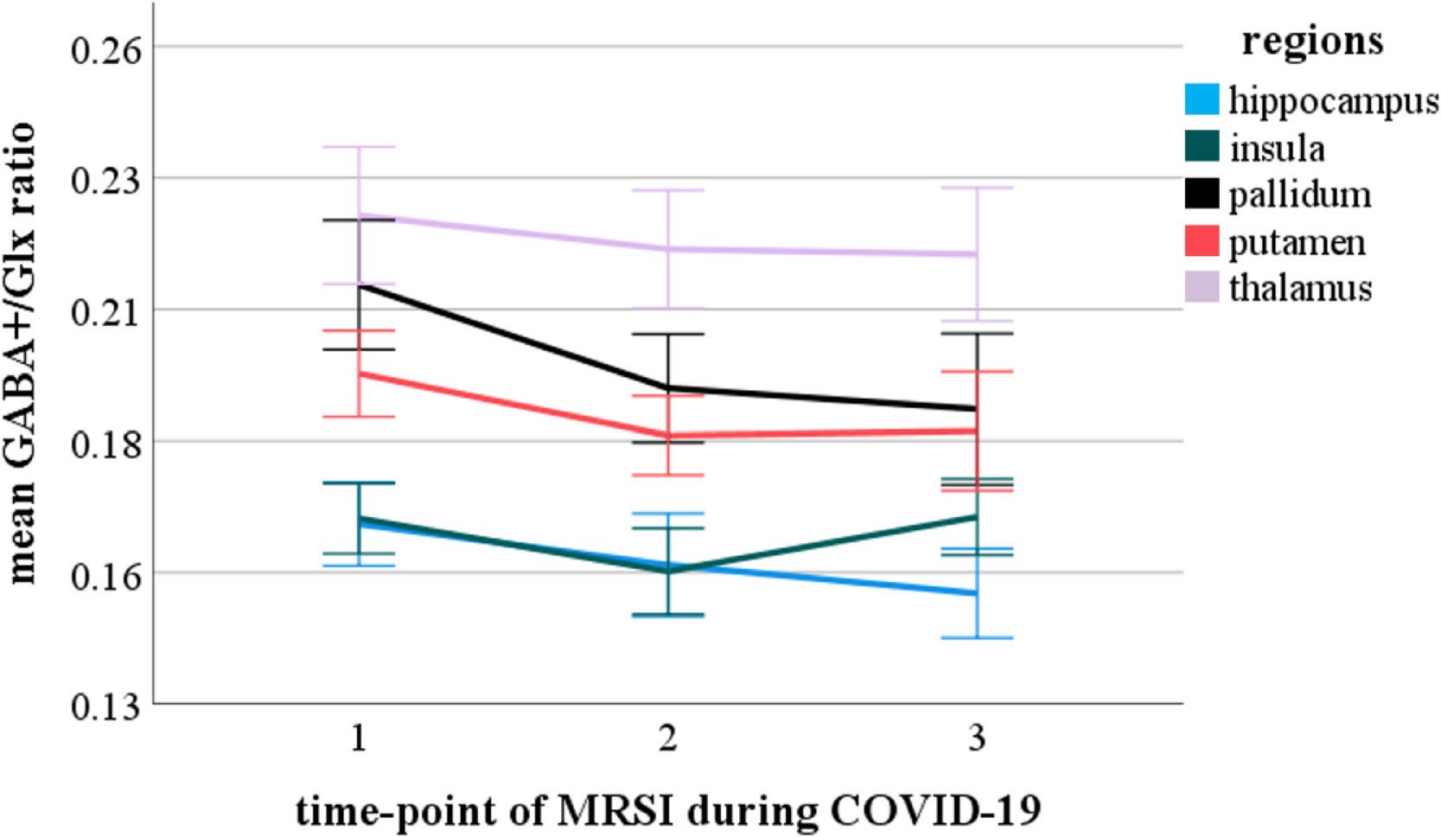
Mean GABA+/Glx ratios for the hippocampus, insula, thalamus, pallidum, and putamen in the whole collective during the COVID-19 pandemic. Error bars indicating SD. GABA+/Glx, gamma-aminobutyric-acid + macromolecules/glutamate + glutamine; SD, standard deviation.

LMMs did not reveal any main effect of time or time*group interaction effects on neurotransmitter ratios in any ROI, after correction for multiple testing (all p_uncorrected_ > 0.01). Linear mixed model analysis revealed a significant main effect of time-point for the pooled Glx/tCr ratio (F(541.2) = 8.2, p_corrected_ < 0.001,) and pooled GABA + /Glx ratio (F(463.7) = 9.7, p_corrected_ < 0.001) for the whole study collective. Post-hoc tests revealed an increase of the Glx/tCr ratio and a decrease of the GABA + /Glx ratio between MRSI-1 and MRSI-2 (Glx/tCr ratio p_corrected_ < 0.001; GABA + /Glx ratio p_corrected_ < 0.001) as well as a decrease in GABA + /Glx between MRSI-1 and MRSI-3 (Glx/tCr ratio p_corrected_ > 0.05; GABA + /Glx ratio p_corrected_ = 0.002). We found neither significant changes between MRSI-2 and MRSI-3, nor an interaction effect of time-point and group as well as time-point and ROI. Separate models for each group depicted a significant main effect of time-point for the Glx/tCr ratio (F(214.9) = 5.3, p_corrected_ = 0.036) in patients with rMDD and ROIs pooled, but not for HI. Post hoc tests showed a significant increase of the Glx/tCr ratio between MRSI-1 and MRSI-2 (p_corrected_ = 0.02) and MRSI-1 and MRSI-3 (p_corrected_ = 0.006). The pooled GABA + /tCr ratio did not change significantly over time in the whole collective or in groups separately (all p_corrected_ > 0.5). Results are graphically illustrated in Figs. 3 and 4 and Supplementary Fig. 1, 2 and 3. Mean neurotransmitter concentrations for ROIs individually and pooled values are reported in Supplementary Table 3 and 4.

### Psychometric variables across COVID-19

**Fig. 5:**
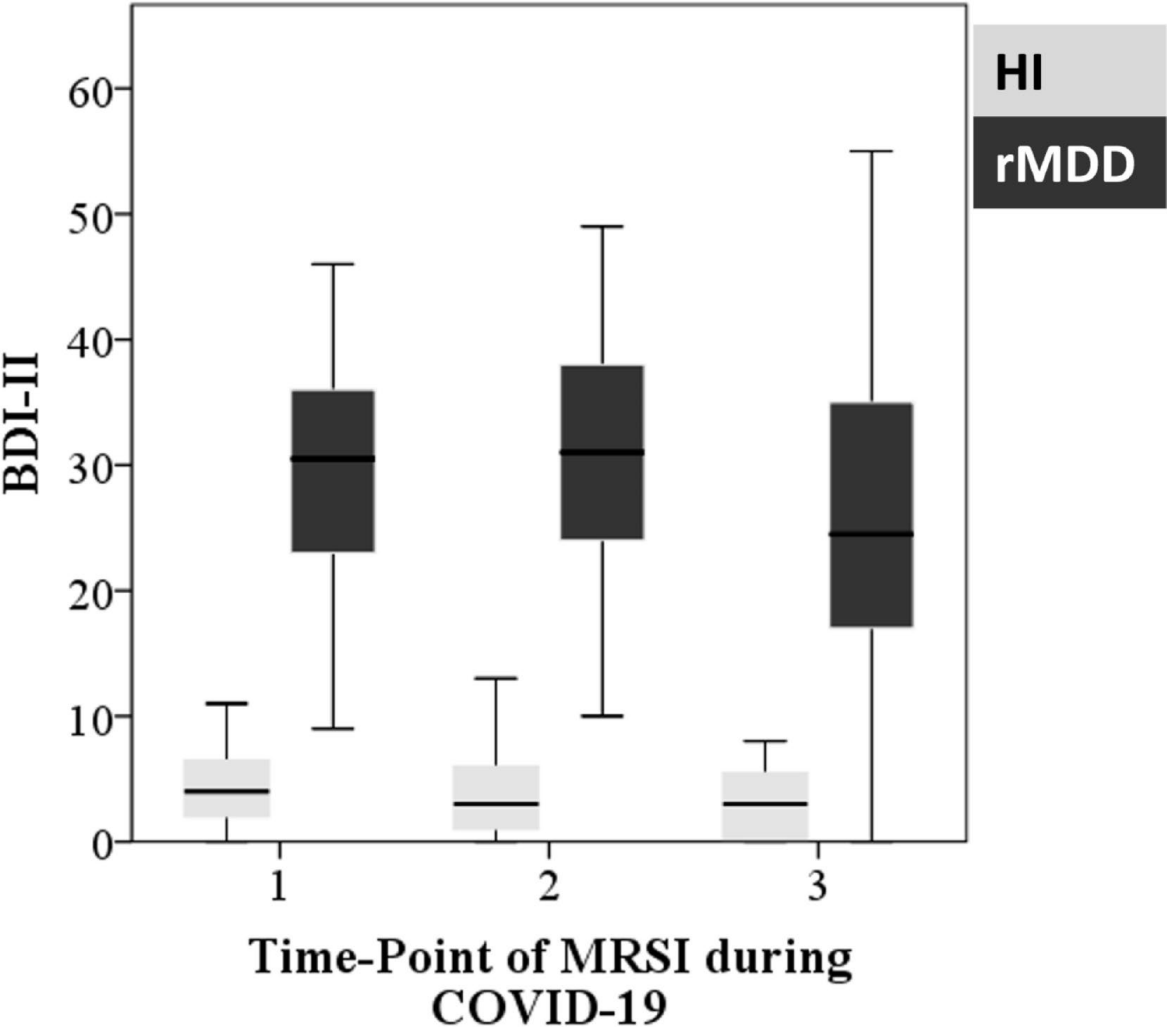
Diagram depicting BDI-II values at each measurement for each group. BDI-II, Beck’s depression inventory II; MRSI, magnetic resonance spectroscopy imaging, rMDD, recurrent major depressive disorder, HI, healthy individuals.

We found no significant main effect of time and no significant interaction between time and group on the BDI-II and ESSI in patients with rMDD and in HI (p_corrected_ > 0.1). We found a main effect of group for BDI-II and ESSI (BDI-II: F(48.8) = 121.4, p_corrected_ < 0.01; ESSI: F(46.3) = 13.7, p_corrected_ < 0.01). In post-hoc t-tests, depressive symptoms were significantly higher in rMDD than in HI (t-test: p_uncorrected_ < 0.001) at each measurement and a significant difference between groups was found regarding the ESSI at MRSI-1 and MRSI-2 (t-test: ESSI-1: p_uncorrected_ < 0.001, ESSI-2: p_uncorrected_ = 0.008). In contrast to HI, patients of the rMDD group presented mean ESSI-levels below the threshold for good social support at every visit. Results are graphically displayed in Fig. 5 and Supplementary Fig. 4.

We found a significant main effect of time of the mean stringency index (p_corrected_ < 0.001, F: 30,444.140). The stringency index gradually increased from MRSI-1 over MRSI-2 to MRSI-3 (for all comparisons between time points p_corrected_ < 0.001).

### Correlations of BDI-II and neurotransmitter ratios

Analysis showed no significant correlations of BDI-II and each neurotransmitter ratio (GABA + /tCr, GABA + /Glx, and Glx/tCr) and ROI separately nor in BDI-II and neurotransmitter ratios of pooled ROIs (all p_corrected_ > 0.05).

## Discussion

To reveal changes of the main excitatory and inhibitory neurotransmitters over the course of the COVID-19 pandemic, we assessed glutamate and GABA ratios in subcortical brain regions and the insula in patients with rMDD and HI. We observed no significant effects of lockdowns on neurotransmitter ratios within any specific ROI in the whole study sample (n = 46) or within groups separately. Analyses for pooled neurotransmitter ratios over all five ROIs yielded a significant increase in Glx/tCr and decrease of GABA + /Glx for the whole collective and within separate analyses for groups an increase in Glx/tCr in patients with rMDD. Stringency index represents an objective marker for social restriction due to COVID-19 pandemic^20^. Although stringency index increased throughout the study period, variables regarding mood (BDI-II) and social support (ESSI) did not change significantly over time. However, the subjective depression severity and the participants’ social support (BDI-II and ESSI) differed between groups.

The COVID-19 pandemic has resulted in increased loneliness^4,5^. Functional and structural imaging data reported a correlate for loneliness within the neuronal regions and circuits involved in coping with social marginalization^22^. In this context, and contrary to our findings, social isolation and lockdowns during the COVID-19 pandemic were found to have an impact on neurobiological parameters. Salomon et al. examined brain structure after the first lockdown and observed an increase in the gray matter volume in the bilateral amygdala, putamen, and anterior temporal cortices in healthy participants, compared to before the COVID-19 pandemic^23^. Also, a positron emission tomography study on patients with neurological disorders reported negative effects of lockdown duration on brain energy metabolism in the sensorimotor cortex, left pyramidal tract, and left amygdala^24^. Contrary, in the present study we found no significant changes in neurotransmitter ratios. Further, we reported no changes in gray matter volume in various brain areas subsequent to COVID-associated lockdowns in a previous analysis by our group^25^. Although the data do not directly address this aspect, an explanation for this finding could be that neurobiological alterations due to COVID-19, as reported previously, are only negligibly mediated by changes of GABA and Glx levels or variations in GABA+/tCr and Glx/tCr might not be reflected in total tissue concentrations. Otherwise, ceiling effects of the first lockdown might be responsible for the lack of changes of GABA and Glx levels subsequent to lockdowns. It is conceivable that the initial lockdown induced substantial neurobiological responses, limiting the capacity for further measurable alterations during later lockdown phases.

In contrast, by pooling neurotransmitter ratios over all ROIs and study groups in an after-the-fact analyses, we observed a significant increase in Glx/tCr as well as a decrease in GABA+/Glx. When separated for groups, only the increase in Glx/tCr in patients with rMDD remained significant, while HIs did not show a significant change. In context with the non-significant changes of symptom severity found in the present analysis, we speculate, that the increase in Glx/tCr ratio may present a neurobiological coping mechanism to prevent the exacerbation of depression. This assumption is based on previous reports that depression is associated with lower levels of glutamate in various brain regions and fast-acting antidepressant medications such as ketamine target the glutamatergic system^13,26^. Another explanation for the elevated Glx/tCr ratio in the rMDD group might relate to the observed differences in social support during COVID-19 pandemic. While the mean social support, measured by ESSI, was good in HI, patients of the rMDD group described insufficient social support during the pandemic. Thus, we hypothesize that adequate social support during times of isolation might prevent changes in neurotransmitter concentrations. Future work is necessary to explore possible glutamate differences in social isolation and depression and its neuropathophysiological bases as well as the preventive potential of social support.

We found no changes in depressive symptom severity over time in the whole collective and in both groups separately, which is in line with a recent meta-analysis^27^. In contrast, surveys in Austria and Germany observed an increase in depression- and anxiety symptoms after strict lockdowns^28,29^. We assume that, maintaining basic life supplies and at least limited contact to close ones during the lockdown might have sufficiently sustained daily routines and social support to prevent changes in mood. In line with this hypothesis, 69.6% (n = 32) of the whole collective reported a good social support, which did not change significantly over the course of the study. Nevertheless, the ESSI differs between groups, illustrating, that patients with rMDD, in contrast to HI, did not have a sufficient social support during COVID-19. In our opinion these results highlight the high ponderosity of stressful and isolation situations as pandemics for patients with rMDD.

### Limitations

Due to the rapid and unexpected effects of the COVID-19 pandemic, the present study differs in various aspects from intervention studies. Specifically, given the unpredictability of the COVID-19 pandemic, we could not assess unified baseline levels before COVID-19, whereas the first lockdown was imposed in March and April of 2020. Normalization of social isolation-induced changes in GMV was reported to occur approximately 90 days after lockdowns^23^. Moreover, brain energy metabolism did not fully recover by 55 days after lifting of social restriction^24^. However, no data is currently available to suggest a time span for the effects of resocialization on neurotransmitter concentrations. Consequently, we cannot project the impact of the first lockdown on glutamate and GABA levels. Although we performed the first measurement around 140 days after the first lockdown, ceiling effects from the first lockdown might have prevented significant findings. Further, we did not find region-specific changes in neurotransmitter levels, which could be a consequence of the sample size, since analyses with pooled brain regions yielded significant differences. Also, patients with rMDD received various psychiatric treatment regimens before and during the study, including pharmacological treatments, electroconvulsive therapy, and transcranial magnetic stimulation. However, non-pharmacological treatments were administered months and years prior to study start and not within the study period. The age was significantly different in both groups, which may impact our data. The reproductive phase of women was not assessed in the study, posing another limitation due to the high percentage of female study subjects and the reported effects of menstrual cycle on GABA and glutamate.

## Conclusion

We demonstrated that COVID-19 associated Lockdowns did not affect brain GABA+/tCr, Glx/tCr and GABA/Glx ratios in specific regions as well as they did not change depressive symptoms in patients with rMDD or HI. This could be attributed to the fact that daily life changes during the COVID-19 pandemic and lockdowns did not affect neurotransmitter levels in researched brain areas or that changes are not detectable by our method and analysis. The severity of depressive symptoms remained unchanged during the whole study period, suggesting that the impact of COVID-19 on depressive symptoms is less than initially thought. This finding contradicts recent studies of the burden of the pandemic on mental health but is in frame with a recent meta-analysis suggesting effects of the pandemic only in certain sub-groups of patients. An explanation for both, the insignificant changes in neurotransmitter levels and depressive symptom severity might be due to a sufficient social support. However, social support was different between HI and patients with rMDD, whereas in patients no adequate social support was observed during the study period. Thus, patients with rMDD might represent a group especially endangered by social restriction measures. Notably, in an after-the-fact analysis of neurotransmitter levels pooled over all ROIs an increase in Glx/tCr and a decrease in GABA+/Glx was observed, however these findings need clarification by further studies addressing the topic.

## Electronic supplementary material

Below is the link to the electronic supplementary material.

Supplementary Material 1

## Data Availability

In frame with data protection guidelines, source data of the study may be requested by the corresponding author Thomas Vanicek with comprehensible reasonability. Data concerning stringency index were taken from “https://github.com/OxCGRT/covid-policy-tracker/blob/master/data/timeseries/stringency_index.csv”.
