# Supplementary material for: Brain glutamate and gamma-aminobutyric acid levels across COVID-19 lockdowns in patients with recurrent major depressive disorder and healthy individuals
Source: Sci Rep. 2025 Jul 1;15:20635. doi: 10.1038/s41598-025-05734-2 (PMC12215143; doi:10.1038/s41598-025-05734-2)
Supplement: Supplementary file 1 — Supplementary Material 1 [file 41598_2025_5734_MOESM1_ESM.pdf]

Brain Glutamate and gamma-aminobutyric acid Levels across COVID-19 Lockdowns in Patients with Recurrent Major Depressive Disorder and Healthy Individuals

Valentin Popper MD†[1,2], Benjamin Spurny-Dworak PhD†[1,2], Jakob Unterholzner MD[1,2], Murray Reed MSc[1,2], Dr. Theresa Wechsler[3], Alexander Kautzky PhD MD[1,2], Peter Stöhrmann MSc[1,2], Manfred Klöbl MSc[1,2], Andreas Mühlberger Prof.[3], Richard Frey Prof. MD[1,2], Dan Rujescu Prof. MD[1,2], Rupert Lanzenberger Prof. MD[1,2], Thomas Vanicek PD MD*[1,2].

† These authors contributed equally to this work and share first authorship

[1]Department of Psychiatry and Psychotherapy, Medical University of Vienna, Vienna, Austria
[2]Comprehensive Center for Clinical Neurosciences and Mental Health, Medical University of Vienna, Vienna, Austria
[3]Department for Psychology, Clinical Psychology and Psychotherapy, University of Regensburg, Regensburg, Germany

Supplementary Tables and Figures

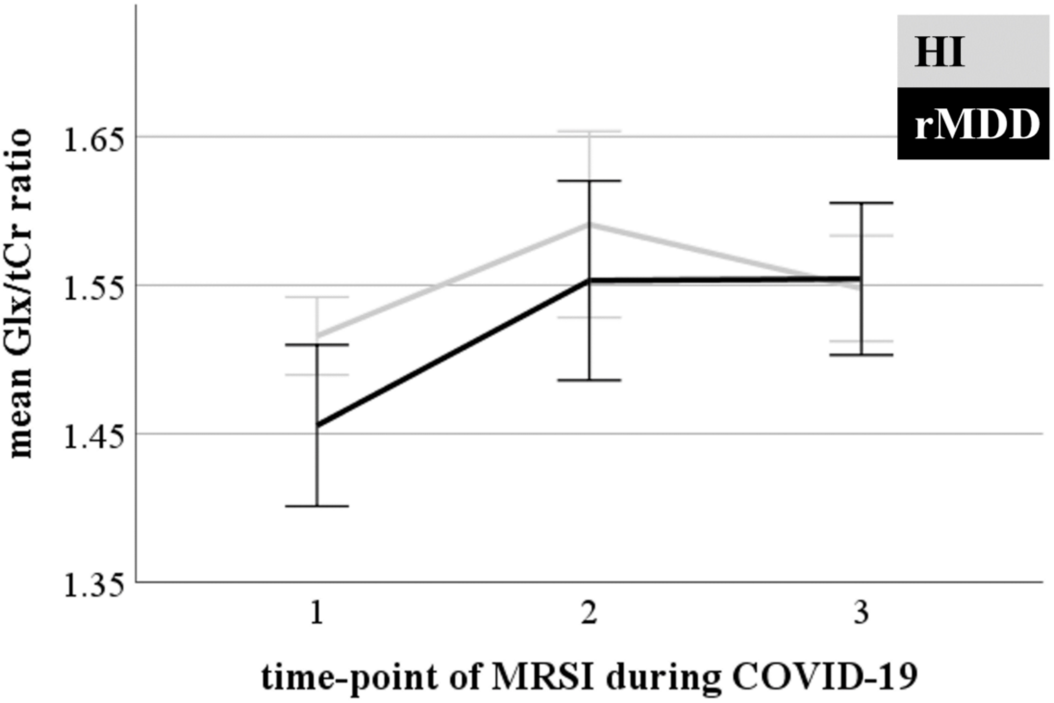

Supplementary Figure 1. *Mean Glx/tCr ratios for all regions pooled in patients with rMDD and HI during the COVID-19 pandemic. Error bars indicating SD. Glx/tCr = glutamate + glutamine/total creatinine; rMDD = recurrent major depressive disorder; HI = healthy individual; SD = standard deviation*

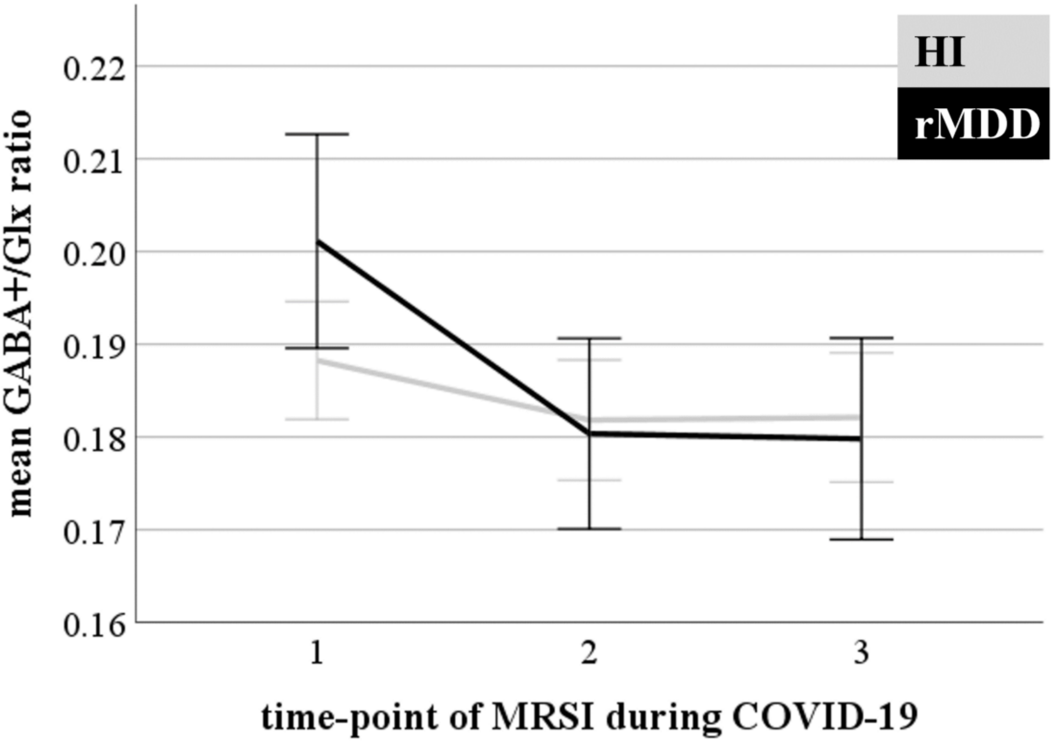

Supplementary Figure 2. *Mean GABA+/Glx ratios for all regions pooled in patients with rMDD and HI during the COVID-19 pandemic. Error bars indicating SD. GABA+/Glx = gamma-aminobutyric-acid + macromolecules/glutamate + glutamine, rMDD = recurrent major depressive disorder; HI = healthy individual; SD = standard deviation*

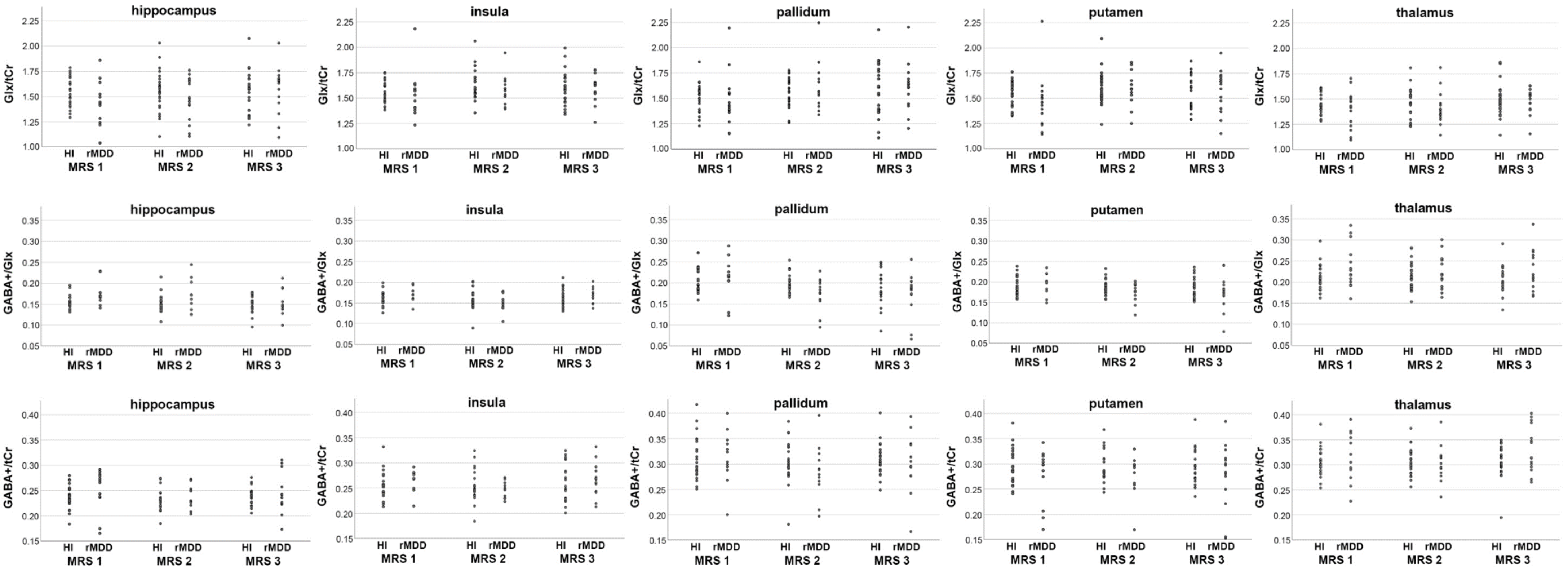

Supplementary Figure 3. *Mean Glx/tCr, GABA+/Glx and GABA+/tCr ratios for the hippocampus, insula, pallidum, putamen and thalamus in HI and rMDD group during the COVID-19 pandemic. Glx = glutamate + glutamine; tCr = total creatinine; GABA+ = gamma-aminobutyric-acid + macromolecules; HI = healthy individuals, rMDD = recurrent Major Depressive Disorder, MRS = magnetic resonance spectroscopy*

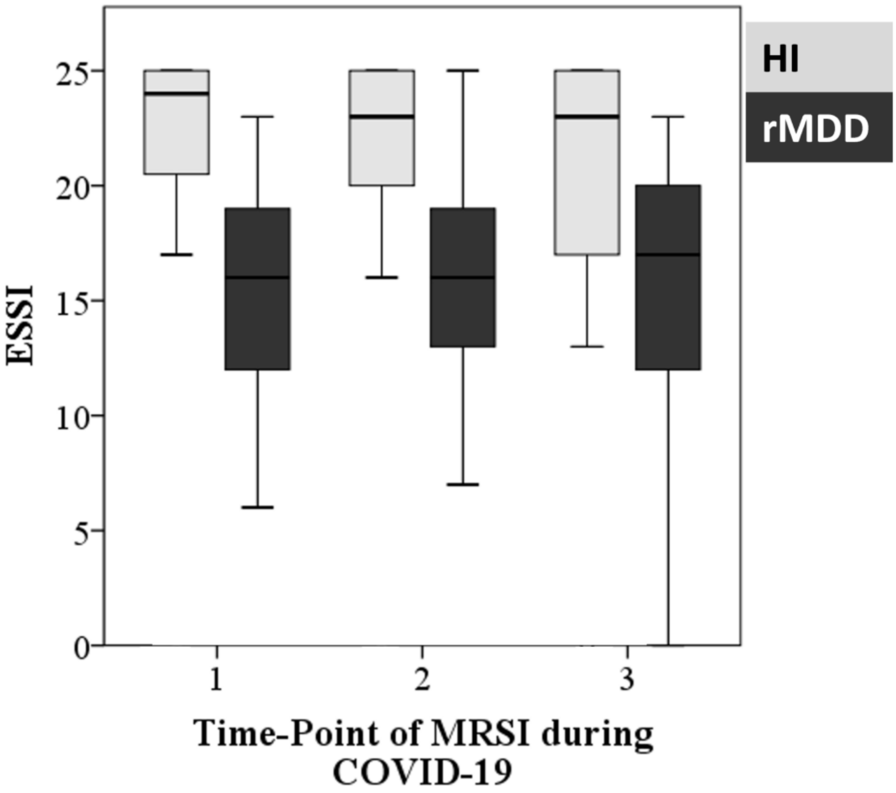

Supplementary Figure 4. *Diagram depicting ESSI values at each measurement for each group. ESSI = Enriched Social Support Inventory, MRSI = magnetic resonance spectroscopy imaging, rMDD = recurrent major depressive disorder, HI = healthy individua*

Supplementary.Tabel.1. *medication in the rMDD (recurrent Major Depressive Disorder) group over the course of the study, as well as current psychotherapy and previous treatment with ECT (electroconvulsive therapy) or ketamine*

**ID**	**Medication at time point 1**	**Medication at time point 2**	**Medication at time point 3**	**Current Psychotherapy**	**previous ECT treatment**	**previous Ketamine-treatment**
**1**	Lamotrigine 100 mg	n.a.	n.a.		x	x
**2**	Quetiapine 25 mg	Quetiapine 25 mg	Quetiapine 25 mg	x		x
**3**	Paroxetine 30 mg	Paroxetine 25 mg	Escitalopram 15 mg, Pregabalin 150 mg	x		
**4**	n.a.	n.a.	n.a.	x		
**5**	Clomipramine 75 mg, Buspirone 10 mg	Clomipramine 75 mg, Buspirone 10 mg	Clomipramine 75 mg, Buspirone 10 mg	x		
**6**	Sertraline 125 mg, Duloxetine 60 mg	Sertraline 125 mg, Duloxetine 60 mg	Sertraline 125 mg, Duloxetine 60 mg			
**7**	Escitalopram 20 mg, Methylphenidate 20 mg, Zolpidem 5 mg,	Escitalopram 20 mg, Methylphenidate 20 mg, Aripiprazole 3 mg, Valdoxan 25 mg	Escitalopram 20 mg, Methylphenidate 20 mg, Aripiprazole 4 mg, Zolpidem 10 mg,			
**8**	Zolpidem 10 mg, Chlorprotixene 100 mg, Tranylcypromine 40 mg	Zolpidem 10 mg, Chlorprotixene 100 mg, Tranylcypromine 40 mg	Zolpidem 10 mg, Chlorprotixene 100 mg, Tranylcypromine 40 mg		x	x
**9**	Levomepromazine 100 mg, Lithium 675 mg, Tranylcypromine 45 mg, Bupropion 450 mg,	Tranylcypromine 30 mg, Bupropion 150 mg, Prothipendyl 160 mg, Aripiprazole 5 mg, Lithium 450 mg	Tranylcypromine 30 mg, Bupropion 150 mg, Prothipendyl 160 mg, Aripiprazole 5 mg, Lithium 450 mg	x	x	x
**10**	n.a.	n.a.	Venlafaxine 300 mg, Mirtazapine 45 mg, Esketamine nasalspray (1/week)			
**11**	n.a.	Trazodone 100 mg	Bupropion 300 mg, Trazodone 150 mg			
**12**	Venlafaxine 150 mg, Mirtazapine 30 mg	Venlafaxine 150 mg, Trazodone 50 mg	Venlafaxine 150 mg, Trazodone 50 mg			
**13**	n.a.	Methylphenidate 30 mg, Aripiprazole 3 mg, Quetiapine 25 mg	Methylphenidate 40 mg, Quetiapine 25 mg			
**14**	Milnacipran 50 mg, Pregabalin 300 mg, Trazodone 100 mg	Milnacipran 100 mg, Pregabalin 350 mg, Trazodone 150 mg	Milnacipran 100 mg, Pregabalin 350 mg, Trazodone 150 mg	x		
**15**	n.a.	n.a.	Venlafaxine 300 mg, Lamotrigine 100 mg			x
**16**	Bupropion 600 mg, Milnacipran 50 mg, Flupentixol/Melitracen	Bupropion 600 mg, Milnacipran 50 mg, Flupentixol/Melitracen	Bupropion 600 mg, Milnacipran 50 mg, Flupentixol/Melitracen			
**17**	n.a.	n.a.	Venlafaxine 75 mg, Quetiapine 25 mg, Methylphenidate 30 mg			
**18**	Sertraline 50 mg, Bupropion 150 mg, Lorazepam 1 mg	Sertraline 50 mg, Bupropion 150 mg, Lorazepam 1 mg	Sertraline 50 mg, Bupropion 150 mg, Lorazepam 1 mg		x	x

Supplementary Table 2. *Comorbidities in the rMDD (recurrent Major Depressive Disorder) group. ICD-10 = International Statistical Classification of Diseases and Related Health Problems, 10th revision.*

Comorbidities in the rMDD group		
**ICD-10 code**	**Diagnosis name**	**Frequency**
F41.0	Panic Disorder	1/18
F40.2	Specific phobias	1/18
F43.1	Post-traumatic stress disorder	1/18
F60.3	Emotionally instable personality disorder	2/18
F60.6	Anxious personality disorder	1/18
F60.7	Dependent personality disorder	1/18
F61	Mixed personality disorder	1/18
F90.0	Disturbance of activity and attention	4/18

Supplementary Table 3. *Mean values of neurotransmitter, ± standard deviation (±SD) in the whole collective as well as in patients with recurrent Major Depressive Disorder (rMDD) and healthy individuals (HI). Values for each region and measurement (MRSI, 1-3) are presented. Glx = glutamate + glutamine; tCr = total creatinine; GABA+ = gamma-aminobutyric-acid + macromolecules;*

		**total collective**			**rMDD group**			**HI group**		
		**MRSI-1**	**MRSI-2**	**MRSI-3**	**MRSI-1**	**MRSI-2**	**MRSI-3**	**MRSI-1**	**MRSI-2**	**MRSI-3**
**hippocampus**	GABA+/tCr	0.24±0.03	0.23±0.02	0.24±0.04	0.25±0.04	0.24±0.02	0.24±0.04	0.24±0.02	0.23±0.02	0.24±0.03
	GABA+/Glx	0.16±0.02	0.16±0.03	0.15±0.02	0.17±0.03	0.17±0.04	0.15±0.03	0.16±0.02	0.15±0.02	0.15±0.02
	Glx/tCr	1.49±0.21	1.58±0.41	1.54±0.22	1.43±0.24	1.48±0.21	1.54±0.24	1.52±0.18	1.65±0.50	1.55±0.21
**insula**	GABA+/tCr	0.26±0.03	0.25±0.03	0.26±0.04	0.26±0.02	0.25±0.02	0.27±0.03	0.26±0.03	0.25±0.03	0.26±0.04
	GABA+/Glx	0.17±0.02	0.16±0.02	0.17±0.02	0.17±0.02	0.15±0.02	0.17±0.02	0.16±0.02	0.16±0.02	0.16±0.02
	Glx/tCr	1.55±0.16	1.69±0.41	1.59±0.15	1.54±0.21	1.64±0.27	1.58±0.13	1.56±0.12	1.73±0.48	1.59±0.17
**putamen**	GABA+/tCr	0.29±0.04	0.29±0.04	0.29±0.05	0.28±0.06	0.28±0.03	0.28±0.07	0.29±0.04	0.29±0.03	0.29±0.04
	GABA+/Glx	0.19±0.02	0.18±0.02	0.18±0.03	0.20±0.03	0.17±0.02	0.17±0.04	0.19±0.02	0.19±0.02	0.19±0.02
	Glx/tCr	1.51±0.20	1.61±0.24	1.57±0.19	1.46±0.27	1.62±0.32	1.58±0.21	1.55±0.13	1.60±0.16	1.55±0.18
**pallidum**	GABA+/tCr	0.31±0.04	0.29±0.06	0.29±0.07	0.31±0.05	0.28±0.08	0.27±0.09	0.31±0.04	0.30±0.04	0.30±0.05
	GABA+/Glx	0.21±0.04	0.19±0.03	0.19±0.04	0.21±0.05	0.17±0.04	0.17±0.05	0.21±0.03	0.20±0.02	0.20±0.04
	Glx/tCr	1.48±0.22	1.57±0.32	1.57±0.29	1.45±0.29	1.63±0.44	1.59±0.36	1.50±0.15	1.53±0.19	1.55±0.23
**thalamus**	GABA+/tCr	0.32±0.04	0.31±0.03	0.32±0.04	0.33±0.06	0.31±0.04	0.33±0.05	0.31±0.03	0.31±0.03	0.31±0.03
	GABA+/Glx	0.22±0.04	0.22±0.04	0.22±0.04	0.24±0.05	0.22±0.04	0.22±0.05	0.21±0.03	0.21±0.03	0.21±0.03
	Glx/tCr	1.43±0.14	1.43±0.15	1.49±0.15	1.40±0.18	1.40±0.15	1.48±0.12	1.44±0.10	1.45±0.15	1.50±0.16

Supplementary Table 4. *Mean values of region of interest (ROI)-pooled neurotransmitter, ± standard deviation (±SD) in the whole collective as well as in patients with recurrent Major Depressive Disorder (rMDD) and healthy individuals (HI) separately. Glx: glutamate + glutamine; GABA+: gamma-aminobutyric-acid + macromolecules; tCr: total creatinine; MRSI: magnetic resonance imaging.*

	whole collective	rMDD	HI
**mean Glx+/tCR MRSI-1**	1.48 (±0.16)	1.43 (±0.18)	1.52 (±0.14)
**mean Glx+/tCR MRSI-2**	1.55 (±0.23)	1.54 (±0.28)	1.55 (±0.18)
**mean Glx+/tCR MRSI-3**	1.55 (±0.19)	1.55 (±0.18)	1.55 (±0.19)
**mean GABA+/Glx MRSI-1**	0.19 (±0.03)	0.19 (±0.04)	0.19 (±0.03)
**mean GABA+/Glx MRSI-2**	0.18 (±0.04)	0.18 (±0.04)	0.18 (±0.03)
**mean GABA+/Glx MRSI-3**	0.18 (±0.04)	0.18 (±0.04)	0.18 (±0.04)
**mean GABA+/tCr MRSI-1**	0.28 (±0.05)	0.29 (±0.06)	0.28 (±0.04)
**mean GABA+/tCr MRSI-2**	0.28 (±0.04)	0.28 (±0.05)	0.28 (±0.04)
**mean GABA+/tCr MRSI-3**	0.28 (±0.05)	0.28 (±0.04)	0.28 (±0.05)
